# Refining genetic associations in multiple sclerosis

**DOI:** 10.1016/S1474-4422(08)70122-4

**Published:** 2008-07

**Authors:** International Multiple Sclerosis Genetics Consortium (IMSGC)

Genome-wide association studies involve several hundred thousand markers and, even when quality control is scrupulous, are invariably confounded by residual uncorrected errors that can falsely inflate the apparent difference between cases and controls (so-called genomic inflation).^1^ As a consequence such studies inevitably generate false positives alongside genuine associations. By use of Bayesian logic and empirical data, the Wellcome Trust Case Control Consortium suggested that association studies in complex disease should involve at least 2000 cases and 2000 controls, at which level they predicted that p values of less than 5×10^−7^ would more commonly signify true positives than false positives.^2^

The screening phase of our recent multiple sclerosis genome-wide association study^3^ involved just 931 trio families and thus fell short of the minimum power recommended by the Wellcome Trust Case Control Consortium. However, the extension phase of our study included 2322 cases, 5418 controls, and 1540 trio families (12 360 individuals in total) and identified three markers exceeding the consortium's threshold—rs6897932 in *IL7R* (p=2·94×10^−7^) and rs12722489 and rs2104286 in *IL2RA* (p=2·96×10^−8^ and 2·16×10^−7^ respectively). These markers showed modest levels of significance in the screening phase of the study (p values 0·0058, 0·0013, and 0·0033, respectively). In overlapping^4^ and independent^5^ data sets, we simultaneously identified association with *IL7R* (rs6897932) through a candidate gene approach. *IL2RA* was suggested as a candidate by its confirmation as a susceptibility gene for type 1 diabetes.^2^ The extensive linkage disequilibrium between rs12722489 and rs2104286 in the *IL2RA* gene meant that it was impossible to determine whether one or other locus exerts a primary effect or whether both influence risk.

**Figure 1:**
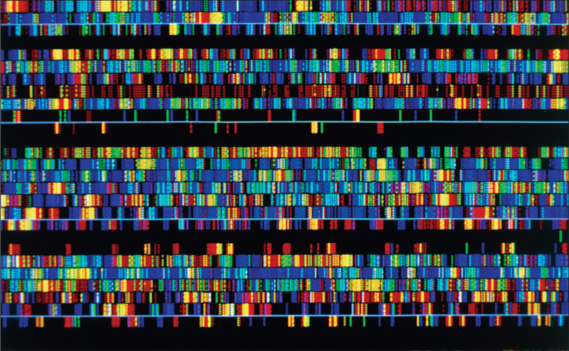
Human DNA sequence

The three identified loci have several similarities. For each the more common (major) allele increases susceptibility, and in each case the risk exerted by this allele is modest (with odds ratios about 1·2). All three of these single-nucleotide polymorphisms have been studied in the HapMap cohorts and curiously in each case the risk allele is even more common in non-white ethnic groups. Because multiple sclerosis is more common in white people than in other ethnic groups, this reverse pattern of allele frequency is a reminder that these alleles account for only a fraction of the heritable influences on susceptibility.

To refine our understanding of these associations, we typed all three variants in an additional 20 708 individuals in Australia, Belgium, Denmark, Finland, France, Germany, Ireland, Italy, the Netherlands, Norway, Sardinia, Spain, Sweden, and new samples from the UK (webappendix). Together with the 12 360 reported in our original screen this provides a total of 33 068 individuals, including 11 019 unrelated cases, 13 616 controls and 2811 trio families (8433 individuals). All individuals involved in this study gave informed consent under appropriate local ethical approval. Overall genotyping efficiency was 98·4% for rs6897932, 95·4% for rs12722489, and 95·7% for rs2104286. None of the three markers showed any significant evidence for deviation from Hardy-Weinberg equilibrium in the controls although deviation was seen in the cases, as expected for genuine associations (webappendix).

In total, 20 population-specific cohorts (14 case-control and six trio family) were considered. Nominally significant association was observed in eight for rs6897932, in nine for rs12722489, and in 13 for rs2104286. In all but three studies, the risk allele as defined in our original screen (ie, the major allele at each locus) was over-represented in cases. None of these three negative findings (Australia and Ireland for rs6897932, and Holland for rs12722489) was significant. In short, all significant studies were in accordance with the original screen and most in which there was no statistically significant association implicated the major allele as expected. Results for the individual studies are shown in the webappendix.

In the control groups, major-allele frequency was 64–77% for rs6897932, 77–90% for rs12722489, and 69–83% for rs2104286. However, applying the Breslow-Day test confirms that there is no evidence of heterogeneity of effect across the populations for any of the markers. Thus, although the frequency of the risk allele shows modest variation between white populations, the effects of these alleles are of undoubted relevance (table).^6^, ^7^

**Table:** Association testing in combined cohorts

	**χ^2^**	**p**	**Odds ratio (95% CI)**
**C allele of rs6897932 (*IL7R*)**			
Case-control*	73·14	1·21×10^−17^	1·200 (1·151–1·252)
Trios†	10·33	1·31×10^−03^	1·153 (1·057–1·258)
**T allele of rs2104286 (*IL2RA*)**			
Case-control*	99·12	2·38×10^−23^	1·247 (1·194–1·302)
Trios†	24·67	6·80×10^−07^	1·278 (1·160–1·409)
**C allele of rs12722489 (*IL2RA*)**			
Case-control*	62·84	2·24×10^−15^	1·234 (1·172–1·300)
Trios†	11.95	5·47×10^−04^	1·232 (1·094–1·387)

* Based on all 14 case-control cohorts taken together but treating each as a separate stratum in a Cochran-Mantel-Haenszel test. In total this analysis includes 11 019 cases and 13 616 controls.

† This analysis is based on all six cohorts of trio families treated together in a transmission-disequilibrium-test analysis. In total this analysis includes 2811 trio families (8433 individuals). Primary statistical analysis was done with PLINK,^6^ and the conditional analysis and genotypic testing was done with UNPHASED.^7^

We confirmed linkage disequilibrium between the two polymorphisms in *IL2RA* (r^2^=0·5). Conditioning on each marker in turn shows that the association seen at rs12722489 is entirely a consequence of its linkage disequilibrium with rs2104286. This finding confirms that rs2104286 (or another single-nucleotide polymorphism in linkage disequilibrium with it) is the primary association even though it showed less significant association than rs12722489 in the original screen. Testing for association at the genotypic level confirms that the homozygous risk genotype confers a significantly greater risk than the heterozygous genotype for both rs6897932 and rs2104286 (webappendix).

This extension analysis illustrates the value of data sets that are significantly larger than the minimum recommended by the Wellcome Trust Case Control Consortium. Although these data convincingly replicate these associations, they do not establish these particular variants as causative. Fine mapping and functional studies will be required.

## Web Extra Material

Webappendix
