# Supplementary material for: Refining genetic associations in multiple sclerosis
Source: Lancet Neurol. 2008 Jul;7(7):567–9. doi: 10.1016/S1474-4422(08)70122-4 (PMC2696028; doi:10.1016/S1474-4422(08)70122-4)
Supplement: Webappendix [file mmc1.pdf]

Samples

Case-Control Cohorts

	Cases	Controls	Power[†] / %
**Australia (AUS)**	472	424	32.4
**Belgium (BEL)**	405	462	31.3
**Denmark (DEN)**	504	541	36.7
**Finland (FIN)**	561	1017	47.3
**Germany (GER)**	206	605	23.2
**Ireland (IRE)**	683	532	41.4
**Italy (ITA)**	863	775	52.8
**Netherlands (NET)**	46	194	9.2
**Norway (NOR)**	738	1027	54.4
**Sardinia (SAR)**	909	576	47.5
**Spain (SPA)**	815	786	51.9
**Sweden (SWE)**	1,100	1,251	67.8
**UK(new)**[*]	1,395	8	-
**UK**	928	2,952	95.0[‡]
**US**	1,394	2,466	84.4
**Total**	11,019	13,616	99.7[§]

[*] The new UK cases include 878 from the Northwest of the UK and 517 from Wales.
[†] This column indicates the power to demonstrate nominally significant association ($p < 5\%$) to a susceptibility allele with Odds Ratio = 1.2 and a background frequency of 80% (assuming that there is total linkage disequilibrium between the tested variant and the causative variant, and a multiplicative model).
[‡] The power for the UK cohort was calculated based on including all samples, new and original.
[§] In the Total row the power to demonstrate highly significant association ($p < 5x10^{-7}$) is shown (the power to demonstrate nominally significant association is 100%).

Trio Family Cohorts

	Trio Families	Power* / %
**Belgium (BEL)**	114	11.8
**Finland (FIN)**	254	20.4
**France (FRA)**	540	37.7
**Sardinia (SAR)**	363	27.1
**UK**	833	53.4
**US**	707†	46.9
**Total**	2,811	96.4

* This column indicates the power to demonstrate nominally significant association ($p < 5\%$) to a susceptibility allele with Odds Ratio = 1.2 and a background frequency of 80% (assuming that there is total linkage disequilibrium between the tested variant and the causative variant and a multiplicative model).

† Eleven of these trios consisted of discordant sib trios for which no parental DNA was available.

NOTE: the data from the German and French Cohorts for all three markers has previously been reported in Genes and Immunity (2008, 9; 259-63) as has the data from the Netherlands (electronically published in April in the same journal). Likewise the data from Sweden and most of the data from Ireland for rs6897932 has previously been reported in Nature Genetics (2007, 38; 1108-13) and the New England Journal of Medicine (2008, 358; 753-4) respectively.

Marker performance in the individual case-control cohorts is shown in the table below

POP	SNP	Missingness / %	HWE_Case / p-value	HWE_Control / p-value
**AUS**	rs6897932	2.90	0.8011	0.6788
**AUS**	rs2104286	2.12	0.6823	0.9055
**AUS**	rs12722489	2.12	0.2451	0.2499
**BEL**	rs6897932	1.50	0.3835	0.3465
**BEL**	rs2104286	0.58	0.1478	0.3455
**BEL**	rs12722489	0.46	0.5801	0.5224
**DEN**	rs6897932	0.86	0.0051	0.8332
**DEN**	rs2104286	0.38	0.7983	0.0257
**DEN**	rs12722489	0.67	1.0000	0.7879
**FIN**	rs6897932	4.12	0.8345	0.7838
**FIN**	rs2104286	0.63	0.5716	0.8561
**FIN**	rs12722489	0.33	1.0000	1.0000
**GER**	rs6897932	0.49	0.6924	0.4526
**GER**	rs2104286	1.11	0.1202	0.4442
**GER**	rs12722489	0.37	0.1534	0.1068
**NED**	rs6897932	0.00	0.0936	0.8674
**NED**	rs2104286	0.00	1.0000	0.8358
**NED**	rs12722489	0.00	0.5699	0.7103
**IRE**	rs6897932	0.16	0.0504	0.9111
**IRE**	rs2104286	0.00	0.1228	0.0665
**IRE**	rs12722489	0.00	1.0000	0.0983
**ITA**	rs6897932	1.71	0.6630	0.5365
**ITA**	rs2104286	1.40	0.8257	0.9105
**ITA**	rs12722489	1.89	0.4354	0.4408
**NOR**	rs6897932	6.06	0.6803	0.8211
**NOR**	rs2104286	3.06	0.8404	0.7097
**NOR**	rs12722489	1.87	0.8962	0.2051
**SAR**	rs6897932	3.10	0.5038	0.7408
**SAR**	rs2104286	1.62	0.3862	0.8785
**SAR**	rs12722489	1.48	0.1814	0.8124
**SPA**	rs6897932	1.75	3.21E-07	0.0002
**SPA**	rs2104286	1.19	0.6409	0.0514
**SPA**	rs12722489	1.25	0.0064	0.1539
**SWE**	rs6897932	3.62	0.2239	0.5391
**SWE**	rs2104286	10.00	0.9329	1.0000
**SWE**	rs12722489	6.76	0.1661	0.1763
**UK**	rs6897932	0.47	0.6889	0.3820
**UK**	rs2104286	3.07	0.0127	0.5564
**UK**	rs12722489	1.80	0.7844	0.3572
**US**	rs6897932	0.28	0.8757	0.3409
**US**	rs2104286	0.78	0.0043	0.1491
**US**	rs12722489	0.03	0.7035	0.6871

HWE = Hardy Weinberg Equilibrium. The significance of any deviation from HWE is shown.

Marker performance in the individual trio family cohorts is shown in the table below

POP	SNP	Missingness / %	HWE_Parents / p-value
**BEL**	rs6897932	0.58	0.4643
**BEL**	rs2104286	0.00	1.0000
**BEL**	rs12722489	0.88	1.0000
**FIN**	rs6897932	1.05	0.3008
**FIN**	rs2104286	3.67	1.0000
**FIN**	rs12722489	3.98	0.6757
**FRA**	rs6897932	1.67	0.4013
**FRA**	rs2104286	3.02	0.5805
**FRA**	rs12722489	2.16	0.7666
**SAR**	rs6897932	1.47	0.6835
**SAR**	rs2104286	3.12	1.0000
**SAR**	rs12722489	3.12	0.8086
**UK**	rs6897932	0.32	0.5203
**UK**	rs2104286	2.80	0.1724
**UK**	rs12722489	2.48	0.4968
**US**	rs6897932	0.42	0.1213
**US**	rs2104286	2.97	0.0071
**US**	rs12722489	1.08	0.1786

HWE = Hardy Weinberg Equilibrium. The significance of any deviation from HWE is shown.

Testing for HWE in the parents is less than ideal since the presence of genuine association is expected to produce a degree of deviation.

The table below shows the number of successful genotypes for each marker as well as the degree of missingness and the evidence for deviation from Hardy Weinberg Equilibrium (HWE) in cases, controls and trio families as combined groups. Although the degree of difference in missingness between cases and controls is highly significant for all three markers the extent of this difference is insufficient to account for the observed associations (even if all failures were confined to a single genotype).

	**rs6897932**	**rs2104286**	**rs12722489**
**Genotypes**			
**- Cases**	10,711	10,488	10,609
**- Controls**	13,475	13,400	13,106
**- Trio Families**	8,363	7,770	7,828
**Missingness[*] / %**			
**- Cases**	2.80	3.39	2.28
**- Controls**	1.04	1.66	1.14
**- Trio Families**	0.83	2.85	2.13
**HWE (p-value)**			
**- Cases**	0.00062	0.00012	0.0085
**- Controls**	0.12	0.18	0.80
**- Trio Parents**	0.43	0.014	0.15

[*] Because some groups did not attempt the typing of each marker in all their available samples the denominator in these calculations varies.

HWE = Hardy Weinberg Equilibrium. The significance of any deviation from HWE is shown.

Results for individual population specific Case Control studies

	RAF_Case / %	RAF_Control / %	p-value	Odds Ratio (CI)
**rs6897932**				
**AUS**	75.6	76.8	0.5728	0.938 (0.752 - 1.171)
**BEL**	77.9	72.8	0.01515	1.317 (1.054 - 1.645)
**DEN**	77.0	71.1	0.002127	1.363 (1.118 - 1.661)
**FIN**	69.7	64.0	0.001943	1.291 (1.098 - 1.517)
**GER**	77.0	74.6	0.3452	1.136 (0.872 - 1.480)
**NET**	76.1	69.1	0.1850	1.425 (0.843 - 2.409)
**IRE**	73.3	73.6	0.8660	0.984 (0.821 - 1.181)
**ITA**	80.4	77.1	0.02226	1.218 (1.028 - 1.443)
**NOR**	74.1	70.7	0.03533	1.184 (1.012 - 1.386)
**SAR**	76.9	74.5	0.1351	1.142 (0.960 - 1.359)
**SPA**	75.4	72.7	0.08045	1.153 (0.983 - 1.352)
**SWE**	74.6	69.8	0.0003386	1.270 (1.114 - 1.447)
**UK**	76.1	72.4	1.69E-05	1.214 (1.111 - 1.327)
**US**	78.1	74.4	0.0002750	1.227 (1.099 - 1.370)
**rs2104286**				
**AUS**	78.1	71.0	0.0006887	1.453 (1.170 - 1.803)
**BEL**	80.9	77.9	0.1192	1.205 (0.952 - 1.524)
**DEN**	77.4	69.5	4.55E-05	1.504 (1.236 - 1.832)
**FIN**	81.6	77.7	0.009948	1.274 (1.059 - 1.531)
**GER**	80.0	75.2	0.04702	1.323 (1.003 - 1.744)
**NET**	80.4	77.8	0.5863	1.171 (0.663 - 2.066)
**IRE**	75.5	69.7	0.003048	1.336 (1.103 - 1.618)
**ITA**	80.8	79.8	0.5019	1.061 (0.892 - 1.263)
**NOR**	75.2	69.6	0.0003095	1.326 (1.137 - 1.547)
**SAR**	86.7	83.4	0.01565	1.292 (1.049 - 1.590)
**SPA**	81.4	77.4	0.005684	1.276 (1.073 - 1.517)
**SWE**	74.8	72.4	0.08226	1.129 (0.984 - 1.296)
**UK**	75.4	71.6	1.73E-05	1.216 (1.112 - 1.330)
**US**	78.3	74.7	0.0002873	1.228 (1.099 - 1.372)
**rs12722489**				
**AUS**	88.6	85.0	0.02249	1.382 (1.046 - 1.826)
**BEL**	89.9	87.4	0.1023	1.285 (0.951 - 1.736)
**DEN**	85.2	80.1	0.001979	1.436 (1.141 - 1.807)
**FIN**	87.5	86.7	0.5411	1.078 (0.848 - 1.369)
**GER**	88.6	85.2	0.08751	1.347 (0.956 - 1.898)
**NET**	84.8	88.9	0.2703	0.694 (0.362 - 1.332)
**IRE**	84.6	80.6	0.01542	1.322 (1.054 - 1.657)
**ITA**	90.2	89.6	0.5127	1.080 (0.858 - 1.359)
**NOR**	82.8	77.6	0.0001834	1.387 (1.168 - 1.648)
**SAR**	93.1	90.1	0.003486	1.487 (1.138 - 1.943)
**SPA**	79.7	79.3	0.8233	1.020 (0.858 - 1.212)
**SWE**	82.1	81.5	0.5990	1.042 (0.894 - 1.216)
**UK**	86.6	83.2	1.15E-06	1.313 (1.176 - 1.465)
**US**	88.0	85.4	0.001235	1.257 (1.094 - 1.444)

RAF = Risk Allele Frequency, CI = 95% Confidence Interval.

Results for individual population specific Trio Family studies

	RAF_Index / %	RAF_Parents / %	p-value	Odds Ratio (CI)
**rs6897932**				
**BEL**	77.2	76.1	0.6506	1.108 (0.711 - 1.728)
**FIN**	69.4	68.9	0.7243	1.051 (0.797 - 1.386)
**FRA**	77.3	75.9	0.07672	1.197 (0.980 - 1.460)
**SAR**	77.2	75.8	0.1482	1.200 (0.937 - 1.537)
**UK**	75.8	74.3	0.05778	1.162 (0.995 - 1.358)
**US**	77.8	76.5	0.1673	1.136 (0.948 - 1.361)
**rs2104286**				
**BEL**	80.3	77.9	0.2159	1.324 (0.848 - 2.066)
**FIN**	81.2	73.8	0.02181	2.834 (1.117 - 7.184)
**FRA**	81.9	78.7	0.0003766	1.465 (1.186 - 1.811)
**SAR**	86.0	85.2	0.1062	1.284 (0.947 - 1.740)
**UK**	75.3	74.2	0.1754	1.118 (0.952 - 1.313)
**US**	78.7	76.5	0.004672	1.314 (1.087 - 1.588)
**rs12722489**				
**BEL**	86.7	86.3	0.7855	1.077 (0.631 - 1.837)
**FIN**	87.7	83.1	0.1088	2.500 (0.784 - 7.968)
**FRA**	89.1	88.4	0.1950	1.194 (0.913 - 1.562)
**SAR**	92.0	91.6	0.4904	1.146 (0.778 - 1.688)
**UK**	86.3	85.0	0.04658	1.220 (1.003 - 1.484)
**US**	87.5	86.2	0.02129	1.297 (1.039 - 1.620)

RAF = Risk Allele Frequency, CI = 95% Confidence Interval.

The results are shown graphically in the following figures.

Figures showing allele frequencies in population specific case-control cohorts

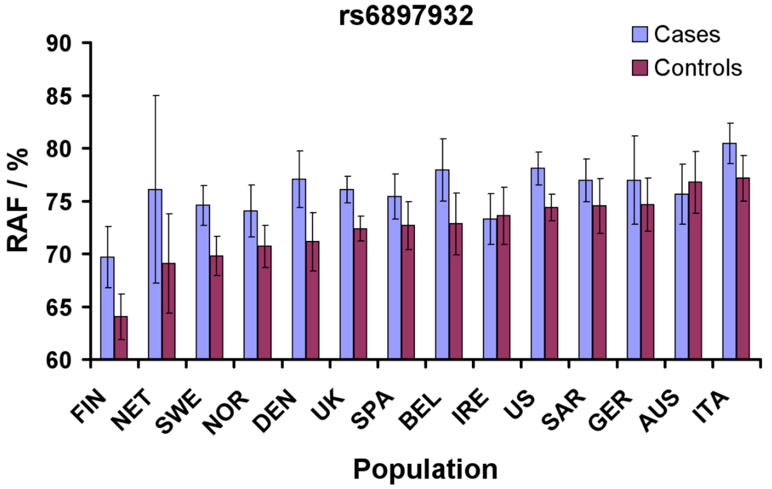

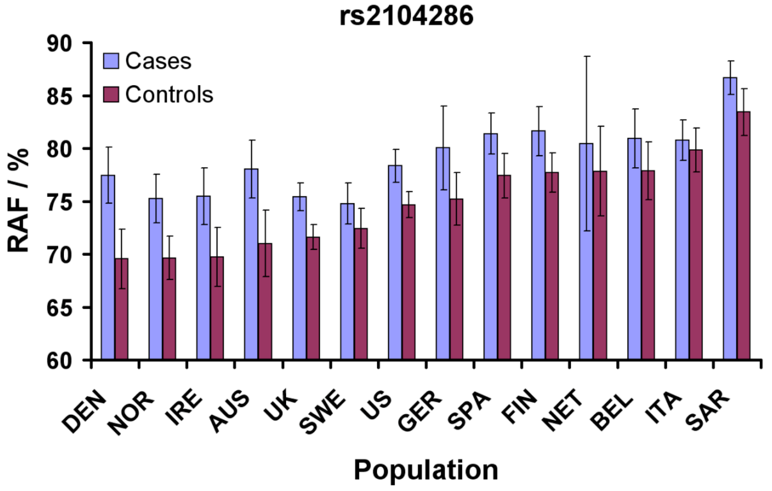

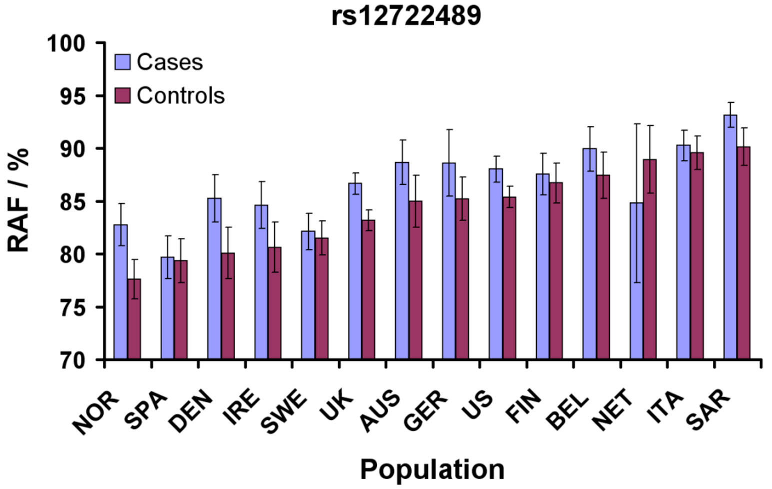

In each figure the populations have been ranked according to the RAF in the controls.

Figures showing allele frequencies in population specific trio family cohorts

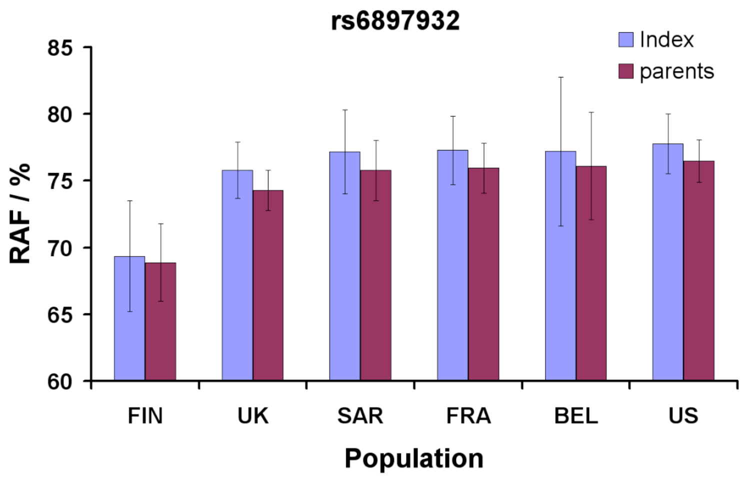

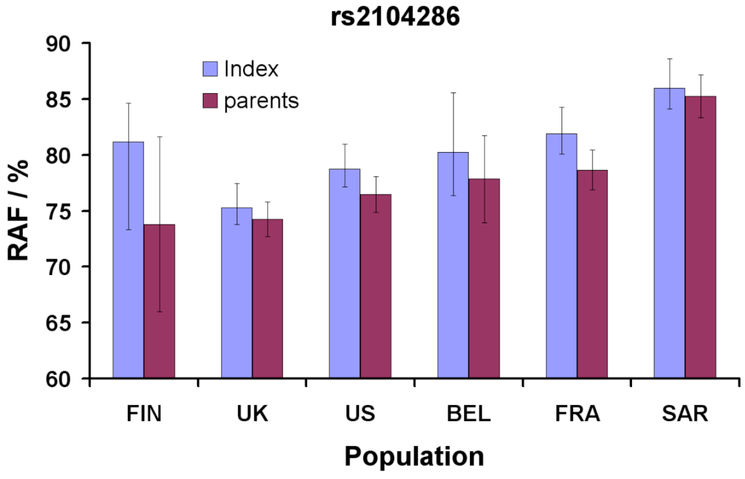

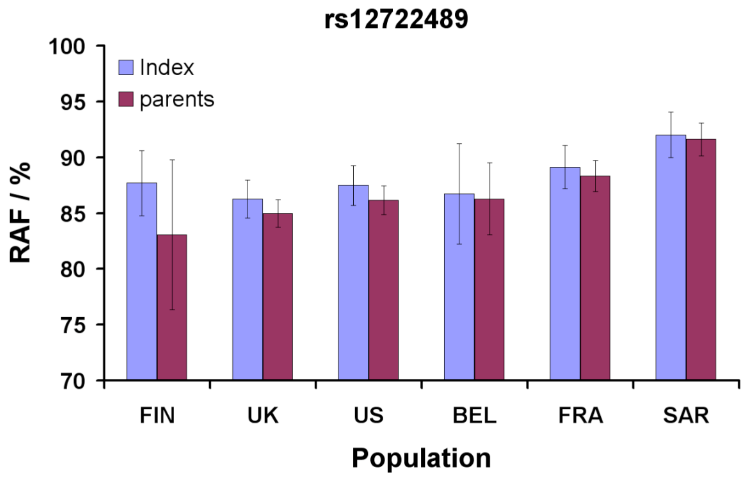

In each figure the populations have been ranked according to the RAF in the controls.

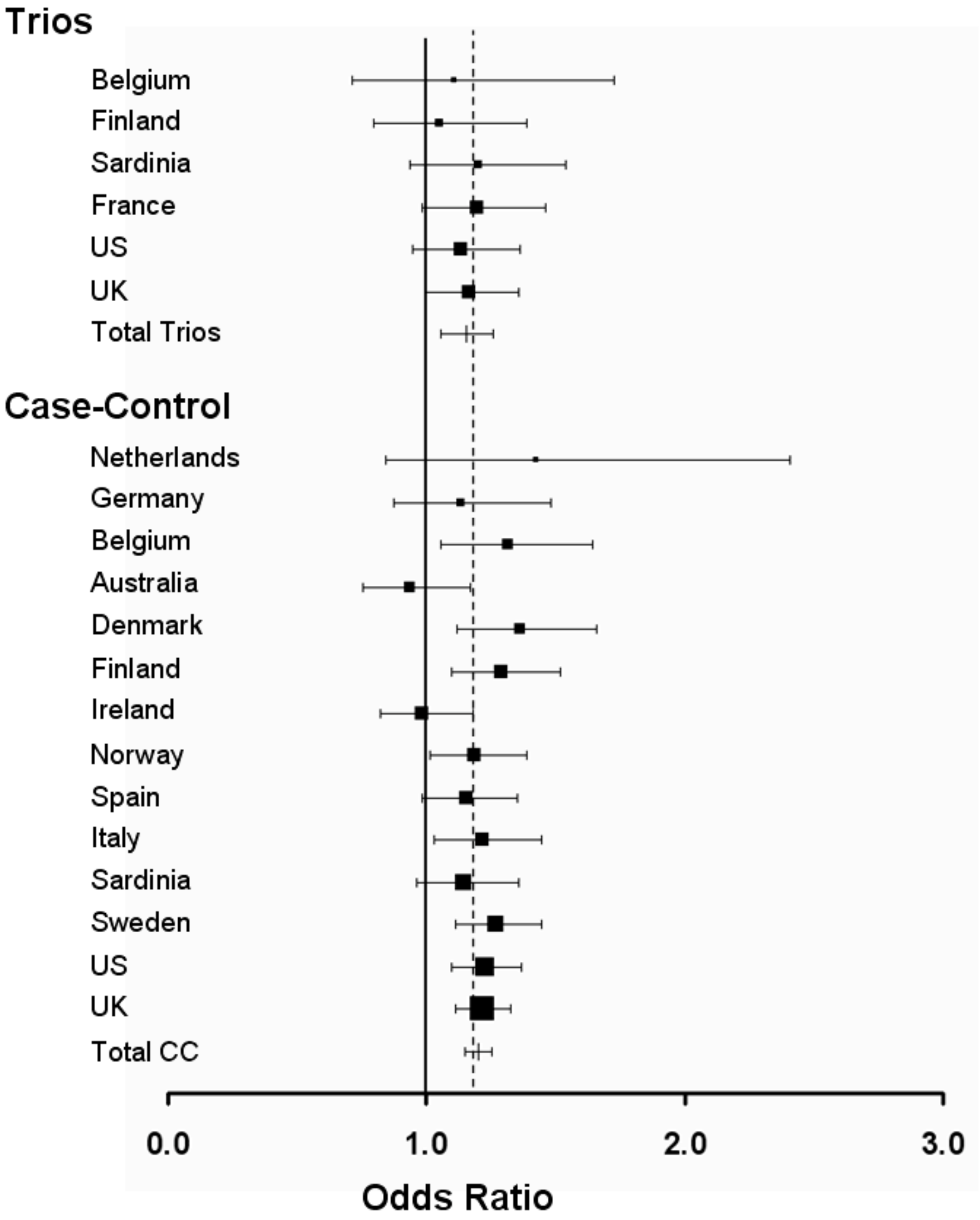

Odds Ratio for the major (C) allele of rs6897932.
Results to the right of the Odds Ratio = 1.0 line are consistent with the original IMSGC screen. The area of the box is proportional to the number of cases included in the respective cohort (no such boxes are included for the analyses based on combined data sets). The dotted line indicates the average Odds Ratio between the combined case-control and combined trio family analyses. Results are plotted in order of sample size.

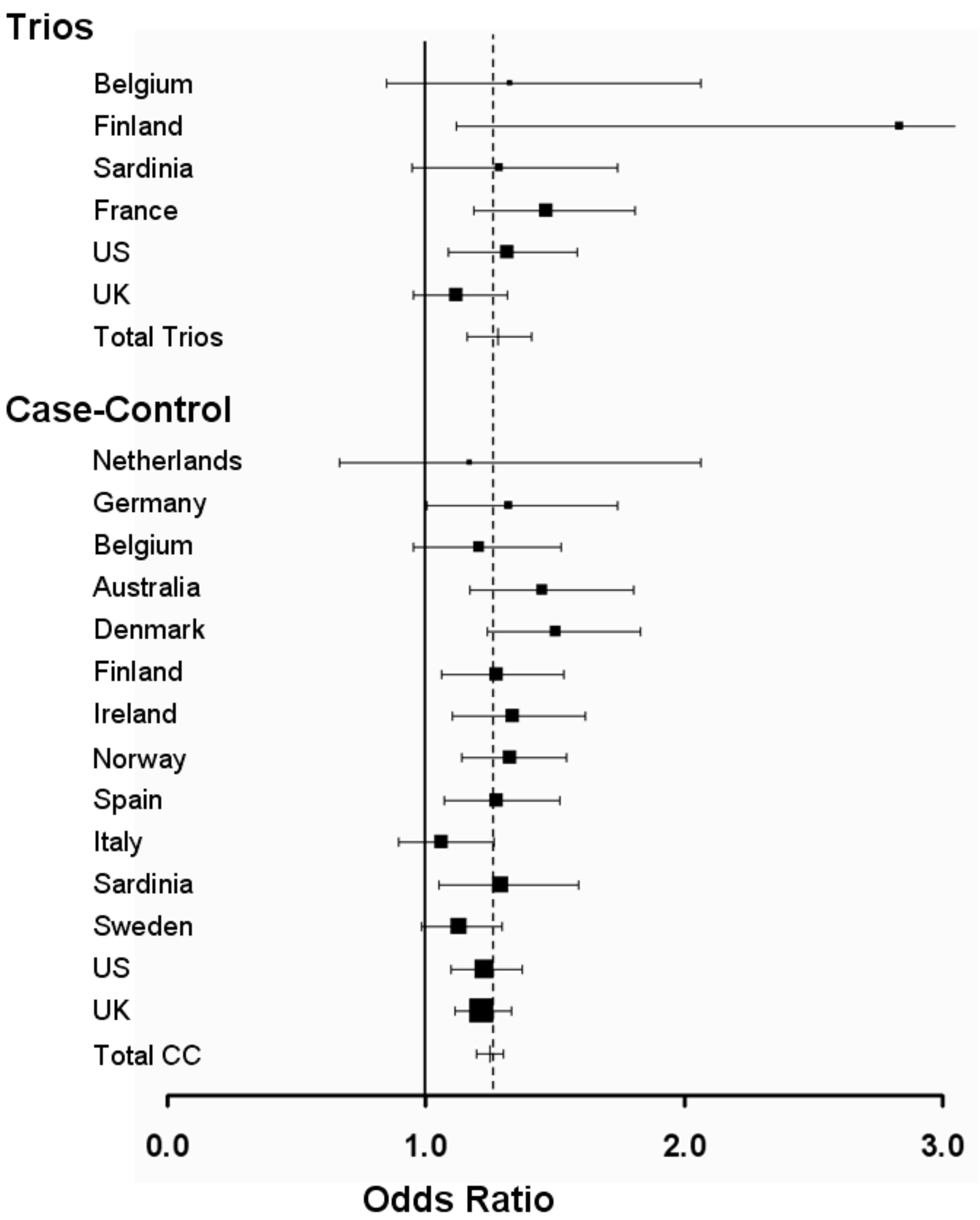

Odds Ratio for the major (T) allele of rs2104286.
Results to the right of the Odds Ratio = 1.0 line are consistent with the original IMSGC screen. The area of the box is proportional to the number of cases included in the respective cohort (no such boxes are included for the analyses based on combined data sets). The dotted line indicates the average Odds Ratio between the combined case-control and combined trio family analyses. Results are plotted in order of sample size. Note only a fraction of the Finnish trios were typed for this marker.

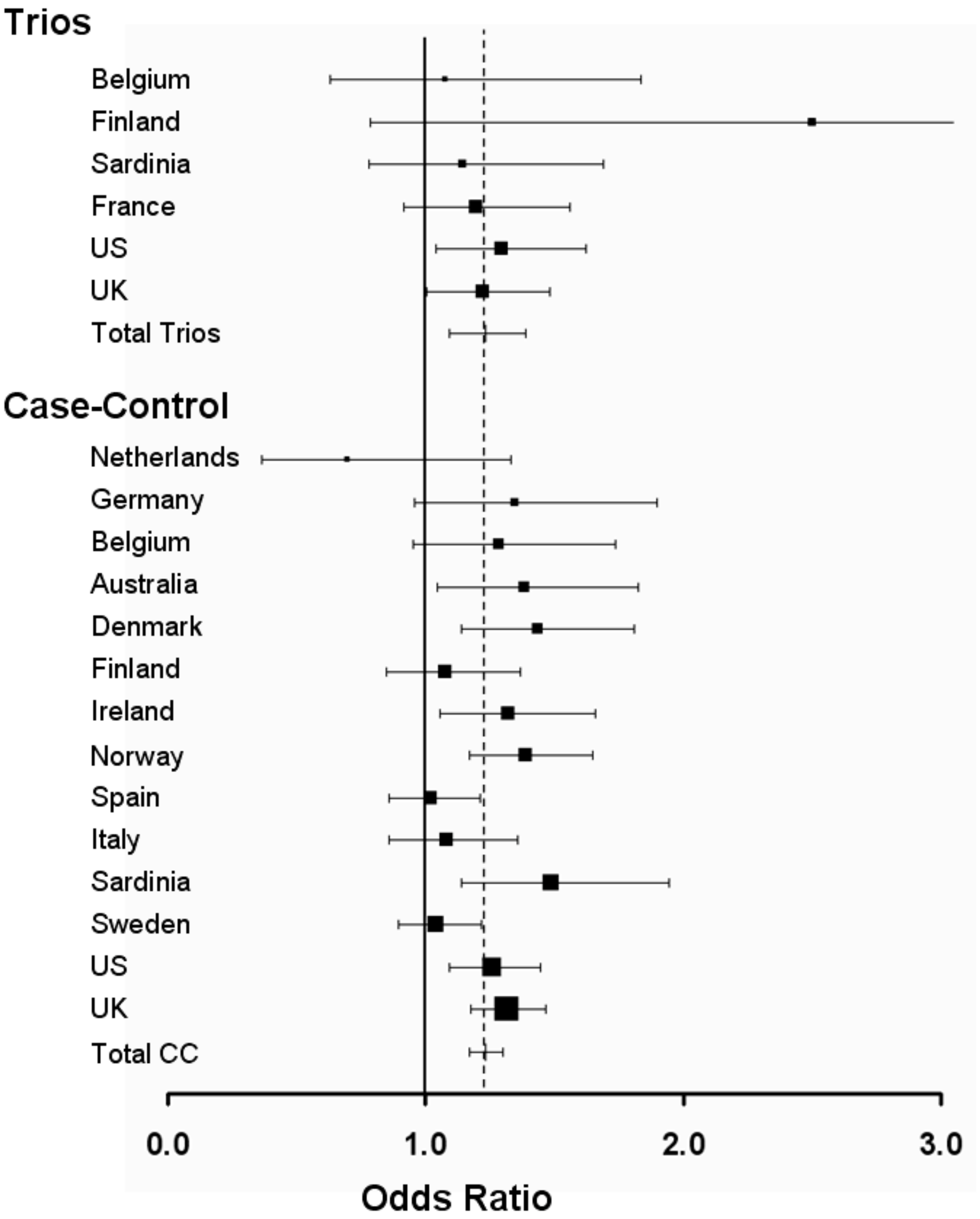

Odds Ratio for the major (C) allele of rs12722489.
Results to the right of the Odds Ratio = 1.0 line are consistent with the original IMSGC screen. The area of the box is proportional to the number of cases included in the respective cohort (no such boxes are included for the analyses based on combined data sets). The dotted line indicates the average Odds Ratio between the combined case-control and combined trio family analyses. Results are plotted in order of sample size. Note only a fraction of the Finnish trios were typed for this marker.

Homogeneity testing
Applying the Breslow-Day test to the 14 case control cohorts confirmed that there is no statistically significant evidence for heterogeneity of effect.

SNP	CHISQ	p-value
rs6897932	14.51	0.34
rs2104286	12.75	0.47
rs12722489	22.83	0.044

Linkage disequilibrium (LD)
The expected LD between rs2104286 and rs12722489 is confirmed

	D' Cases	D' Controls	r^2 Cases	r^2 Controls
Case-Control	0.917	0.930	0.475	0.477
Trios	1.000	0.994	0.517	0.486

Does rs12722489 exert any effect independent of rs2104286?
In order to determine whether the signal from rs12722489 was just a reflection of LD with rs2104286 conditional analyses were performed using UNPHASED.

There is no evidence for any allelic effect from rs12722489 independent of rs2104286.

	Chisq	p-value
Case-Control	0.7848	0.38
Trios	0.1818	0.67

And no evidence that rs12722489 interacts with rs2104286 to influence risk

	Chisq	p-value
Case-Control	0.0484	0.83
Trios	4.559	0.033

These data indicate that the signal from rs12722489 is entirely a reflection of its LD with rs2104286. For completeness the reverse conditioning shows that rs2104286 does exert an allelic effect after correcting for rs12722489.

	Chisq	p-value
Case-Control	53.28	2.90E-13
Trios	16.04	0.000062

Genotypic testing

Table Genotypic testing in the 14 case control cohorts combined

	Chi2*	p-value	Genotype	Odds Ratio (CI)
**rs6897932**	87.4	1.05E-19	C/C	1.390 (1.255-1.539)
**(IL7R)**			C/T	1.115 (1.004-1.238)
			T/T	Reference
**rs2104286**	123.5	1.53E-27	T/T	1.476 (1.322-1.648)
**(IL2RA)**			C/T	1.124 (1.003-1.259)
			C/C	Reference
**rs12722489**	74.36	7.11E-17	C/C	1.351 (1.139-1.602)
**(IL2RA)**			C/T	1.054 (0.884-1.256)
			T/T	Reference

CI = 95% confidence interval

* A 2 degree of freedom test
